# Association between the C-reactive protein to albumin ratio and unplanned readmission in ulcerative colitis: insights from a cohort study

**DOI:** 10.3389/fmed.2026.1715011

**Published:** 2026-02-11

**Authors:** Junyi Zhan, Tianqi Wang, Xiaobin Zhao, Jiaqi Zhu, Shiyu Ji, Yujie Zhao, Dongli Wang

**Affiliations:** 1 Department of Gastroenterology, Hangzhou Hospital of Traditional Chinese Medicine, Hangzhou, Zhejiang, China; 2 First Clinical Medical College, Shandong University of Traditional Chinese Medicine, Jinan, Shandong, China; 3 Department of Gastroenterology, Affiliated Hospital of Shandong University of Traditional Chinese Medicine, Jinan, Shandong, China

**Keywords:** albumin, cohort study, C-reactive protein, ulcerative colitis, unplanned readmission

## Abstract

**Objective:**

This study aimed to investigate the association between the C-reactive protein to albumin ratio (CAR) and unplanned readmissions in patients with ulcerative colitis (UC) and to evaluate its potential value as a predictive indicator.

**Methods:**

This study included 412 patients with UC who were hospitalized at the Affiliated Hospital of Shandong University of Traditional Chinese Medicine between June 2017 and June 2024. Cox proportional hazards models were used to evaluate the relationship between CAR, C-reactive protein (CRP), albumin (ALB), and unplanned readmission in patients with UC. Kaplan-Meier survival curves were plotted to analyze the differences in unplanned readmission rates across different value ranges. Restricted Cubic Splines (RCS) were employed to explore the dose-response relationship between these three variables and unplanned readmissions. Additionally, a subgroup analysis was conducted to evaluate the applicability of the model across different populations. The predictive performance of CAR was assessed using Receiver Operating Characteristic analysis.

**Results:**

During the 1-year follow-up, the unplanned readmission rate among patients with UC was 27.43%. After adjusting for potential confounders, each 1-unit increase in CAR was associated with a 126.9% higher risk of unplanned readmission. Kaplan-Meier survival curves demonstrated significant differences in unplanned readmission rates among UC patients stratified by CAR, CRP, and ALB quartiles (log-rank test, *P* < 0.001). The RCS curves revealed a positive correlation (*P* for overall < 0.001) and a non-linear relationship (*P* for non-linear < 0.001) between CAR and unplanned readmission rates in patients with UC. Threshold effect analysis identified an inflection point for unplanned readmissions in the regression model (*W* = 0.654). Subgroup analysis suggested a potential interaction between hypertension and CAR in relation to unplanned readmission in patients with UC. Finally, the CAR demonstrated good predictive performance at the 1-month, 3-month, 6-month, and 1-year follow-up periods, with the area under the receiver operating characteristic curve values of 0.813, 0.779, 0.778, and 0.799, respectively.

**Conclusion:**

Elevated CAR levels were significantly correlated with increased rates of unplanned readmissions, suggesting its potential as an independent prognostic indicator for patients with UC.

## 1 Introduction

Ulcerative colitis (UC) is a chronic, recurrent inflammatory bowel disease (1, 2). Global epidemiological data from 2023 indicates approximately 5 million people worldwide suffer from UC, with incidence rates continuing to rise globally (1). Current clinical treatments for UC, such as anti-inflammatory drugs, immunosuppressants, and biologics, can effectively alleviate clinical symptoms. However, due to the chronic, protracted, and recurrent nature of the disease, patients still face a significant long-term disease burden and reduced quality of life (3, 4).

The annual readmission rate for UC patients reaches as high as 46% (5, 6). Unplanned readmission serves as a critical indicator for assessing disease control quality, typically suggesting inadequate disease management or suboptimal care following initial discharge. This not only exacerbates the health burden on patients but is also significantly associated with increased mortality risk, prolonged hospitalization duration, and higher healthcare costs (6, 7). Consequently, there is an urgent need to identify clinical indicators with strong predictive value for unplanned readmission in UC patients.

C-reactive protein (CRP) is closely associated with mucosal inflammation severity and disease activity, serving as a vital biomarker for UC disease monitoring and management (8–10). Albumin (ALB) is a commonly used laboratory indicator for assessing nutritional status and chronic inflammation severity (11). Low albumin levels are strongly correlated with the risk of colectomy (12), disease recurrence (13), and mortality (14). The C-reactive protein to albumin ratio (CAR) simultaneously reflects inflammation, nutritional status, and immune function, and is considered a more stable and sensitive prognostic assessment tool than either single indicator alone (15, 16). Recent studies indicate that CAR has been used to evaluate UC disease severity (17) and predict treatment response (18), demonstrating promising clinical application potential.

Current research on the relationship between CAR and readmission risk in UC patients remains limited. Therefore, this study, based on a large single-center cohort with a 7-year follow-up, aims to investigate the association between CAR and unplanned readmission in UC patients and evaluate its predictive value.

## 2 Materials and methods

### 2.1 Study participants

This retrospective cohort study utilized data from patients hospitalized for UC at the Affiliated Hospital of Shandong University of TCM between June 2017 and June 2024. All participants were followed up for 1 year through telephone interviews, outpatient visits, and inpatient medical records.

The inclusion criteria were as follows: (1) age ≥ 18 years; (2) diagnosis in accordance with the American College of Gastroenterology guidelines (19); and (3) availability of CRP and ALB measurements. The exclusion criteria were as follows: (1) age < 18 years; (2) comorbid severe cardiopulmonary disease, hepatic insufficiency, or renal dysfunction; (3) current malignancy; (4) concomitant non-IBD immune disorders; and (5) prior colectomy.

**FIGURE 1:**
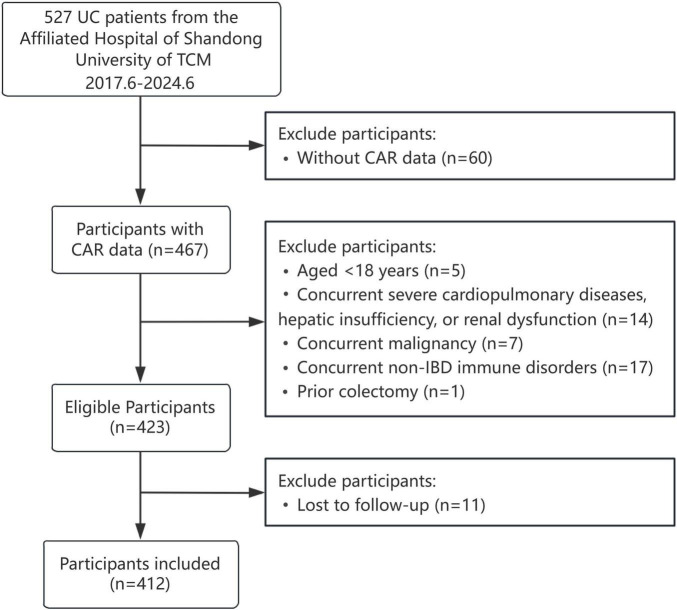
Participant selection process flowchart.

Of the 527 participants initially screened, 115 were excluded based on the above criteria, yielding a final analytical cohort of 412 individuals, as detailed in Figure 1.

The study was conducted in accordance with the principles of the Declaration of Helsinki and was approved by the ethics committee of the Affiliated Hospital of Shandong University of TCM (Approval No. 2024-152-ky). Written informed consent was obtained from all participants or their legal guardians at the time of initial hospitalization.

### 2.2 Definition of CAR and unplanned readmission

CAR was defined as the ratio of serum CRP (mg/L) to serum albumin (g/L).

Unplanned readmission referred to any unscheduled hospitalization at our institution caused by acute exacerbation of UC or inadequate clinical management (20). Planned admissions for follow-up surveillance, enteral nutrition, or scheduled medication infusion were excluded. Each case of suspected unplanned readmission was independently assessed by two senior physicians, each with over 10 years of clinical experience. Any discrepancies in evaluation were resolved through discussion until a consensus was reached.

### 2.3 Covariates

Several clinical characteristics were assessed in this study, including demographics, comorbidities, medication use, and laboratory tests. Demographic data consisted of sex, age, smoking history, and alcohol use. comorbidities included hypertension, diabetes, and cardiovascular disease. Medication use encompassed 5-aminosalicylic acid (5-ASA), immunosuppressants, probiotics, and glucocorticoids (GCS). Laboratory tests involved hemoglobin, platelet count (PLT), white blood cell count (WBC), alanine aminotransferase (ALT), aspartate aminotransferase (AST), total bilirubin, CRP, and ALB. Clinical data were independently collected and cross-verified by two trained administrators.

### 2.4 Statistical analysis

All statistical analyses were conducted using R 4.3.2 and DecisionLine 1.0. Continuous variables were expressed as mean ± standard deviation if normally distributed, or as median (IQR) if not. Categorical variables were summarized as frequencies and percentages (%). Group comparisons were carried out using Student’s *t*-test for normally distributed continuous variables and the Mann-Whitney U test for non-normally distributed variables. For categorical variables, either Pearson’s chi-square test or Fisher’s exact test was applied, depending on expected frequencies.

Cox proportional hazards models were employed to evaluate the associations among CAR, CRP, and ALB levels and unplanned readmission in UC patients. Differences in readmission rates across quartiles of each variable were evaluated using Kaplan-Meier survival curves and compared with the log-rank test. Restricted Cubic Splines (RCS) was used to explore potential dose-response relationships between these three variables and unplanned readmission. The likelihood ratio test was applied to assess non-linearity and identify potential threshold effects. For subgroup analyses, patients were stratified by sex, age, smoking history, alcohol use, comorbidities, and medication use, and interaction effects were assessed. The predictive performance of CAR, CRP, and ALB for unplanned readmission was evaluated using receiver operating characteristic (ROC) curves and the area under the curve (AUC). A two-tailed *P* < 0.05 was considered statistically significant.

## 3 Results

### 3.1 Baseline characteristics

This study included 412 participants with a mean age of 46.59 ± 13.78 years, comprising 247 men and 165 women. The median CRP level was 6.00 (15.85) mg/L, and the mean albumin level was 38.67 ± 5.46 g/L. Compared with patients without unplanned readmission, those with unplanned readmission tended to have higher CRP and lower ALB (Supplementary Figure 1). There were no significant intergroup differences in the history of 5-ASA, biologics, or immunosuppressants. The use of probiotics gradually decreased with increasing CAR quartile. The use of GCS gradually increased with increasing CAR quartile. During the 1-year follow-up period, 113 unplanned readmission events occurred, corresponding to a rate of 27.43%. Among patients with UC who experienced unplanned readmission, worsening disease severity was the primary cause, accounting for 105 (92.92%) cases. No mortality was observed during the follow-up period.

As shown in Table 1, participants were stratified into four groups according to quartiles of the CAR: Q1: < 0.063; Q2: 0.063–0.157; Q3: 0.157–0.459; Q4: > 0.459. A statistically significant trend toward increased unplanned readmission rates was observed with ascending CAR quartiles (*P* < 0.05). Patients with high CAR levels may exhibit a higher prevalence of hypertension, a greater proportion of disease activity, and increased bowel movement frequency, particularly in the severe bowel movement frequency group, where the proportion significantly increases. Concurrently, they may present with elevated PLT, WBC, and AST levels, along with reduced hemoglobin and albumin levels. Furthermore, patients with a high CAR may exhibit elevated CRP and total bilirubin levels.

**TABLE 1:** Baseline characteristics.

Characteristics	CAR				*P-*value
	<0.063 (*N* = 103)	0.063–0.157 (*N* = 103)	0.157–0.459 (*N* = 103)	>0.459 (*N* = 103)	
Age (yr)	47.53 ± 13.32	45.18 ± 14.47	49.42 ± 14.72	44.23 ± 12.07	0.003
Sex, *n* (%)					0.879
Female	60 (58.25)	60 (58.25)	62 (60.19)	65 (63.11)	
Male	43 (41.75)	43 (41.75)	41 (39.81)	38 (36.89)	
Alcohol, *n* (%)					0.994
No	93 (90.29)	94 (91.26)	93 (90.29)	93 (90.29)	
Yes	10 (9.71)	9 (8.74)	10 (9.71)	10 (9.71)	
Hypertension, *n* (%)					0.01
No	92 (89.32)	98 (95.15)	87 (84.47)	99 (96.12)	
Yes	11 (10.68)	5 (4.85)	16 (15.53)	4 (3.88)	
Smoke, *n* (%)					0.481
No	95 (92.23)	88 (85.44)	92 (89.32)	92 (89.32)	
Yes	8 (7.77)	15 (14.56)	11 (10.68)	11 (10.68)	
Diabetes, *n* (%)					0.49
No	100 (97.09)	101 (98.06)	99 (96.12)	97 (94.17)	
Yes	3 (2.91)	2 (1.94)	4 (3.88)	6 (5.83)	
Heart disease, *n* (%)					0.303
No	97 (94.17)	98 (95.15)	99 (96.12)	102 (99.03)	
Yes	6 (5.83)	5 (4.85)	4 (3.88)	1 (0.97)	
Clinical typing, *n* (%)					0.875
new-onset	14 (13.59)	13 (12.62)	17 (16.50)	15 (14.56)	
Recurrent type	89 (86.41)	90 (87.38)	86 (83.50)	88 (85.44)	
Colonoscopy examination, *n* (%)					0.034
Relief period	9 (8.74)	7 (6.80)	6 (5.83)	0 (0.00)	
Active phase	94 (91.26)	96 (93.20)	97 (94.17)	103 (100.00)	
Increased defecation, *n* (%)					<0.001
Normal	32 (31.07)	24 (23.30)	16 (15.53)	10 (9.71)	
Mild	29 (28.16)	28 (27.18)	36 (34.95)	13 (12.62)	
Moderate	33 (32.04)	27 (26.21)	26 (25.24)	27 (26.21)	
Severe	9 (8.74)	24 (23.30)	25 (24.27)	53 (51.46)	
Hematochezia condition, *n* (%)					0.349
Normal	13 (12.62)	14 (13.59)	17 (16.50)	11 (10.68)	
Mild	36 (34.95)	35 (33.98)	30 (29.13)	24 (23.30)	
Moderate	47 (45.63)	45 (43.69)	42 (40.78)	52 (50.49)	
Severe	7 (6.80)	9 (8.74)	14 (13.59)	16 (15.53)	
Use of 5-ASA, *n* (%)					0.092
No	20 (19.42)	24 (23.30)	25 (24.27)	12 (11.65)	
Yes	83 (80.58)	79 (76.70)	78 (75.73)	91 (88.35)	
Use of immunosuppressive agents, *n* (%)					0.457
No	100 (97.09)	97 (94.17)	101 (98.06)	98 (95.15)	
Yes	3 (2.91)	6 (5.83)	2 (1.94)	5 (4.85)	
Use of probiotics, *n* (%)					0.019
No	61 (59.22)	66 (64.08)	61 (59.22)	45 (43.69)	
Yes	42 (40.78)	37 (35.92)	42 (40.78)	58 (56.31)	
Use of GCS, *n* (%)					<0.001
No	94 (91.26)	88 (85.44)	79 (76.70)	63 (61.17)	
Yes	9 (8.74)	15 (14.56)	24 (23.30)	40 (38.83)	
Mucosal biopsy, *n* (%)					<0.001
Normal	6 (5.83)	1 (0.97)	0 (0.00)	0 (0.00)	
Mild	35 (33.98)	28 (27.18)	33 (32.04)	14 (13.59)	
Moderate	51 (49.51)	56 (54.37)	49 (47.57)	50 (48.54)	
Severe	11 (10.68)	18 (17.48)	21 (20.39)	39 (37.86)	
Extent of disease, *n* (%)					0.1
E1	36 (34.95)	32 (31.07)	32 (31.07)	20 (19.42)	
E2	32 (31.07)	37 (35.92)	38 (36.89)	33 (32.04)	
E3	35 (33.98)	34 (33.01)	33 (32.04)	50 (48.54)	
Use of biologics, *n* (%)					0.879
No	100 (97.09)	99 (96.12)	98 (95.15)	98 (95.15)	
Yes	3 (2.91)	4 (3.88)	5 (4.85)	5 (4.85)	
Platelet count (×10^9^/L)	251.00 (91.00)	248.00 (115.00)	269.00 (119.00)	316.00 (166.00)	<0.001
Hemoglobin (g/L)	129.00 (22.00)	128.00 (28.00)	134.00 (29.00)	118.00 (36.00)	<0.001
White blood cell count (×10^9^/L)	5.57 (2.30)	5.51 (2.54)	6.62 (2.76)	7.45 (4.66)	<0.001
Alanine aminotransferase (U/L)	14.00 (9.00)	14.00 (9.00)	14.00 (13.00)	12.00 (15.00)	0.592
Aspartate aminotransferase (U/L)	17.00 (7.00)	17.00 (8.00)	18.00 (8.00)	15.00 (8.00)	<0.001
Total bilirubin (μmoI/L)	12.40 (6.10)	10.90 (6.50)	10.70 (6.40)	9.70 (4.00)	<0.001
C-reactive protein (mg/L)	1.30 (1.00)	3.60 (1.50)	9.90 (7.20)	31.20 (27.30)	<0.001
Albumin (g/L)	40.83 ± 3.99	38.47 ± 5.02	39.70 ± 5.02	35.68 ± 6.24	<0.001
C-reactive protein to albumin ratio	0.03 (0.03)	0.10 (0.05)	0.26 (0.16)	0.85 (0.84)	<0.001
Readmission, *n* (%)					<0.001
No	95 (92.23)	90 (87.38)	75 (72.82)	39 (37.86)	
Yes	8 (7.77)	13 (12.62)	28 (27.18)	64 (62.14)	

5-ASA, 5-aminosalicylic acid; GCS, glucocorticoids.

Supplementary Table 1 presents the characteristics of UC patients grouped by unplanned readmission status within 1 year of follow-up. Compared to those without unplanned readmission, UC patients who experienced unplanned readmission had significantly higher WBC counts, PLT counts, AST levels, CRP levels, and CAR values, as well as lower albumin levels (*P* < 0.05). In addition, they exhibited a higher rate of GCS use.

### 3.2 Association between the CAR and unplanned readmission in UC patients

As shown in Table 2, a significant association was observed between the CAR and unplanned readmission in UC patients across all three models (*P* < 0.001). In the fully adjusted Model 3, each unit increase in the CAR was associated with a 126.9% increase in the risk of unplanned readmission. When participants were categorized by CAR quartiles, those in the Q3 and Q4 groups showed significantly higher risks of unplanned readmission compared to the Q1 group, with a statistically significant trend across quartiles (*P* < 0.001).

**TABLE 2:** The association between CAR and unplanned readmission.

	Variables	Model 1		Model 2		Model 3	
		HR(95%CI)	*P-*value	HR(95%CI)	*P*-value	HR(95%CI)	*P*-value
CAR	CAR	2.24 (1.94, 2.60)	<0.001	2.27 (1.94, 2.64)	<0.001	2.27 (1.88, 2.75)	<0.001
	CAR (Quartile)						
		Q1	Reference		Reference		Reference	
	Q2	1.66 (0.69, 4.02)	0.257	1.68 (0.69, 4.04)	0.251	1.32 (0.53, 3.24)	0.552
	Q3	3.79 (1.73, 8.31)	0.001	3.77 (1.72, 8.28)	0.001	3.14 (1.40, 7.04)	0.005
	Q4	11.86 (5.68, 24.77)	<0.001	12.02 (5.74, 25.17)	<0.001	11.38 (5.21, 24.88)	<0.001
	*P* for trend		<0.001		<0.001		<0.001
CRP	CRP	1.03 (1.02, 1.03)	<0.001	1.03 (1.02, 1.03)	<0.001	1.03 (1.02, 1.03)	<0.001
	CRP (Quartile)						
		Q1	Reference		Reference		Reference	
	Q2	1.21 (0.52, 2.80)	0.656	1.22 (0.53, 2.82)	0.648	1.08 (0.46, 2.54)	0.862
	Q3	2.94 (1.42, 6.06)	0.004	2.92 (1.41, 6.03)	0.004	2.85 (1.36, 5.96)	0.005
	Q4	9.18 (4.71, 17.90)	<0.001	9.30 (4.76, 18.17)	<0.001	8.43 (4.11, 17.26)	<0.001
	*P* for trend		<0.001		<0.001		<0.001
ALB	ALB	0.94 (0.91, 0.97)	<0.001	0.93 (0.90, 0.97)	<0.001	0.94 (0.90, 0.98)	0.002
	ALB (Quartile)						
		Q1	Reference		Reference		Reference	
	Q2	0.51 (0.31, 0.82)	0.006	0.49 (0.30, 0.80)	0.005	0.47 (0.27, 0.80)	0.005
	Q3	0.37 (0.22, 0.63)	<0.001	0.36 (0.21, 0.61)	<0.001	0.40 (0.22, 0.71)	0.002
	Q4	0.43 (0.26, 0.70)	0.001	0.41 (0.24, 0.68)	0.001	0.48 (0.26, 0.90)	0.023
	*P* for trend		<0.001		< 0.001	0.013	<0.001

Model 1: no covariates were adjusted. Model 2: age and sex were adjusted. Model 3: age, sex, smoking, drinking, hypertension, diabetes, heart disease, 5-aminosalicylic acid drug, glucocorticoid agents, immunosuppressive agents, probiotics drug, platelet, hemoglobin, white cell count, ALT, AST, total bilirubin, blood urea nitrogen, and creatinine were adjusted.

Table 3 presents the associations of CRP and ALB with unplanned readmission in UC patients. CRP was significantly associated with unplanned readmission across all models (*P* < 0.001). In the fully adjusted Model 3, each 1 mg/L increase in CRP was associated with a 2.7% increase in readmission risk. Similarly, ALB was also significantly associated with unplanned readmission across all models (*P* < 0.05). In Model 3, each 1 g/L increase in ALB was associated with a 6.1% decrease in readmission risk. When analyzed by quartiles, both CRP and ALB exhibited significant trends for readmission risk (*P* < 0.05). Supplementary Table 2 presents the final Cox proportional hazards model for the association between CAR and unplanned readmission in UC patients.

**TABLE 3:** Threshold effect analysis of CAR.

Outcome	Unplanned readmission
Line effect	2.14 (1.76, 2.60) < 0.001
Inflection point (W)	0.654
<W segment effect 1	46.66 (17.57, 123.95) < 0.001
>W segment effect 2	1.41 (1.08, 1.83) 0.011
Log likelihood ratio	<0.001

HR, hazard ratio; CI, 95% confidence interval, and *P*-value.

**FIGURE 2:**
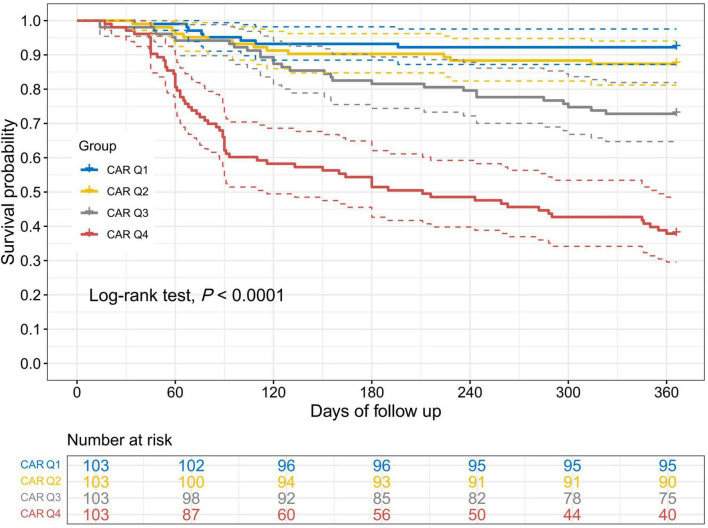
Kaplan Meier survival curve analysis of unplanned readmission rate in patients with UC based on CAR quartiles. CAR, C-reactive protein to albumin ratio. Kaplan-Meier curves stratified by CAR quartiles showed a clear gradient, with higher CAR associated with lower readmission-free survival over 1 year.

Kaplan-Meier survival curves demonstrated significant differences in unplanned readmission rates among groups stratified by CAR, CRP, and ALB quartiles over follow-up time (Log-rank test, *P* < 0.001). Specifically, higher readmission rates were observed in the high CAR group (Figure 2), high CRP group (Supplementary Figure 2A), and low ALB group (Supplementary Figure 2B).

### 3.3 RCS regression and threshold effect analysis

**FIGURE 3:**
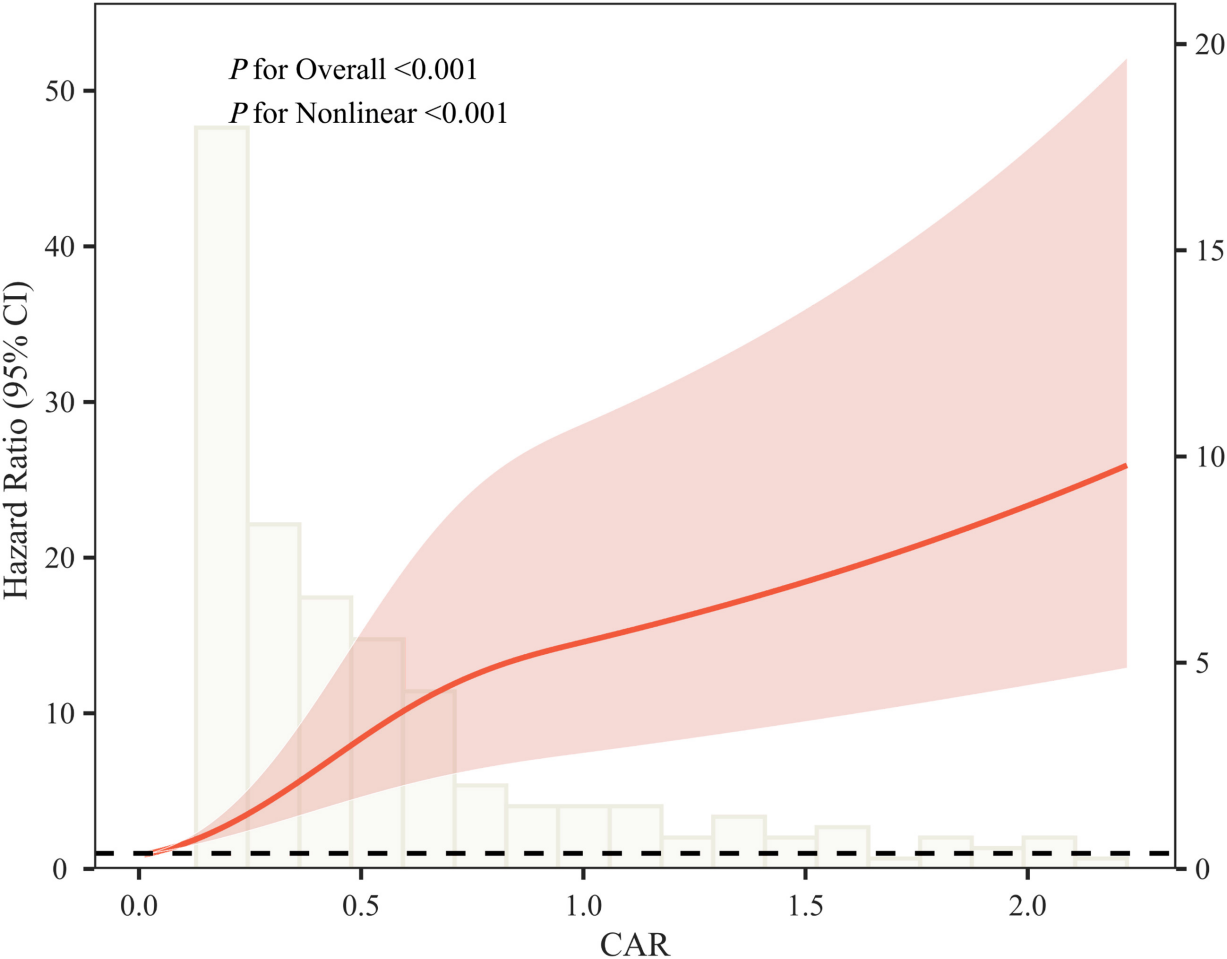
Triple spline analysis of CAR and unplanned readmission risk within 1 year after discharge in patients with ulcerative colitis. CAR, C-reactive protein to albumin ratio. Spline analysis demonstrated a significant, non-linear dose-response relationship between CAR and the 1-year risk of unplanned readmission. The risk changed more steeply at lower CAR levels and continued to vary at higher values (*P* for overall < 0.001; *P* for non-linear < 0.001).

The RCS curve revealed a positive correlation between the CAR and unplanned readmission in UC patients (*P* for overall < 0.001), with a significant non-linear relationship (*P* for non-linear < 0.001) (Figure 3).

Threshold effect analysis identified an inflection point at 0.654 in the regression model for unplanned readmission in UC. Using a piecewise Cox regression model, effect sizes were calculated on either side of the inflection point. To the left of the inflection point, each unit increase in CAR was associated with a marked increase in the risk of unplanned readmission (HR = 46.66, 95% CI: 17.57-123.95, *P* < 0.001). In contrast, beyond the inflection point, the effect of CAR on readmission risk was attenuated (HR = 1.41, 95% CI: 1.08–1.83, *P* = 0.011) (Table 3).

Supplementary Figures 3A, B present the RCS curves illustrating the associations of CRP and ALB with unplanned readmission in UC patients. CRP exhibited a non-linear positive correlation with readmission, while ALB showed a linear negative correlation.

### 3.4 Subgroup analysis and model evaluation

As shown in Table 4, subgroup analysis suggested a potential interactive effect between hypertension and CAR on unplanned readmission in UC patients (*P* for interaction = 0.011). Specifically, hypertensive individuals exhibited a significantly higher risk of unplanned readmission, where each unit increase in CAR conferred an 8.14-fold increase in the risk (HR = 8.14, 95% CI: 2.81–23.62, *P* < 0.001). In other subgroups, the association between CAR and unplanned readmission remained generally consistent.

**TABLE 4:** Subgroup analysis of CAR on unplanned readmission in UC.

Variable	HR(95%CI)	*P*-value	*P* for interaction
Overall	2.24 (1.94, 2.60)	<0.001	
Sex			0.26
Female	2.43 (1.97, 2.99)	<0.001	
Male	2.14 (1.71, 2.66)	<0.001	
Age 60			0.26
No	2.17 (1.85, 2.55)	<0.001	
Yes	4.02 (2.33, 6.94)	<0.001	
Smoke			0.823
No	2.21 (1.87, 2.60)	<0.001	
Yes	2.21 (1.55, 3.16)	<0.001	
Alcohol			0.876
No	2.23 (1.91, 2.59)	<0.001	
Yes	2.40 (1.37, 4.20)	0.002	
Hypertension			0.011
No	2.22 (1.91, 2.59)	<0.001	
Yes	8.14 (2.81, 23.62)	<0.001	
Diabetes			0.843
No	2.25 (1.93, 2.63)	<0.001	
Yes	2.62 (1.27, 5.41)	0.009	
Heart disease			0.974
No	2.23 (1.93, 2.59)	<0.001	
Yes	1.90 (0.01, 682.14)	0.83	
Use of 5-ASA			0.681
No	2.04 (1.47, 2.81)	<0.001	
Yes	2.30 (1.93, 2.74)	<0.001	
Use of GCS			0.379
No	2.39 (1.96, 2.91)	<0.001	
Yes	1.89 (1.48, 2.42)	<0.001	
Use of immunosuppressive agents			0.747
No	2.26 (1.95, 2.62)	<0.001	
Yes	1.84 (0.62, 5.42)	0.271	
Use of probiotics			0.899
No	2.27 (1.87, 2.76)	<0.001	
Yes	2.21 (1.74, 2.81)	<0.001	
Use of biologics			
No	2.41 (2.07, 2.81)	<0.001	0.369
Yes	1.40 (0.23, 8.40)	0.714	

5-ASA, 5-aminosalicylic acid; GCS, glucocorticoids.

**FIGURE 4:**
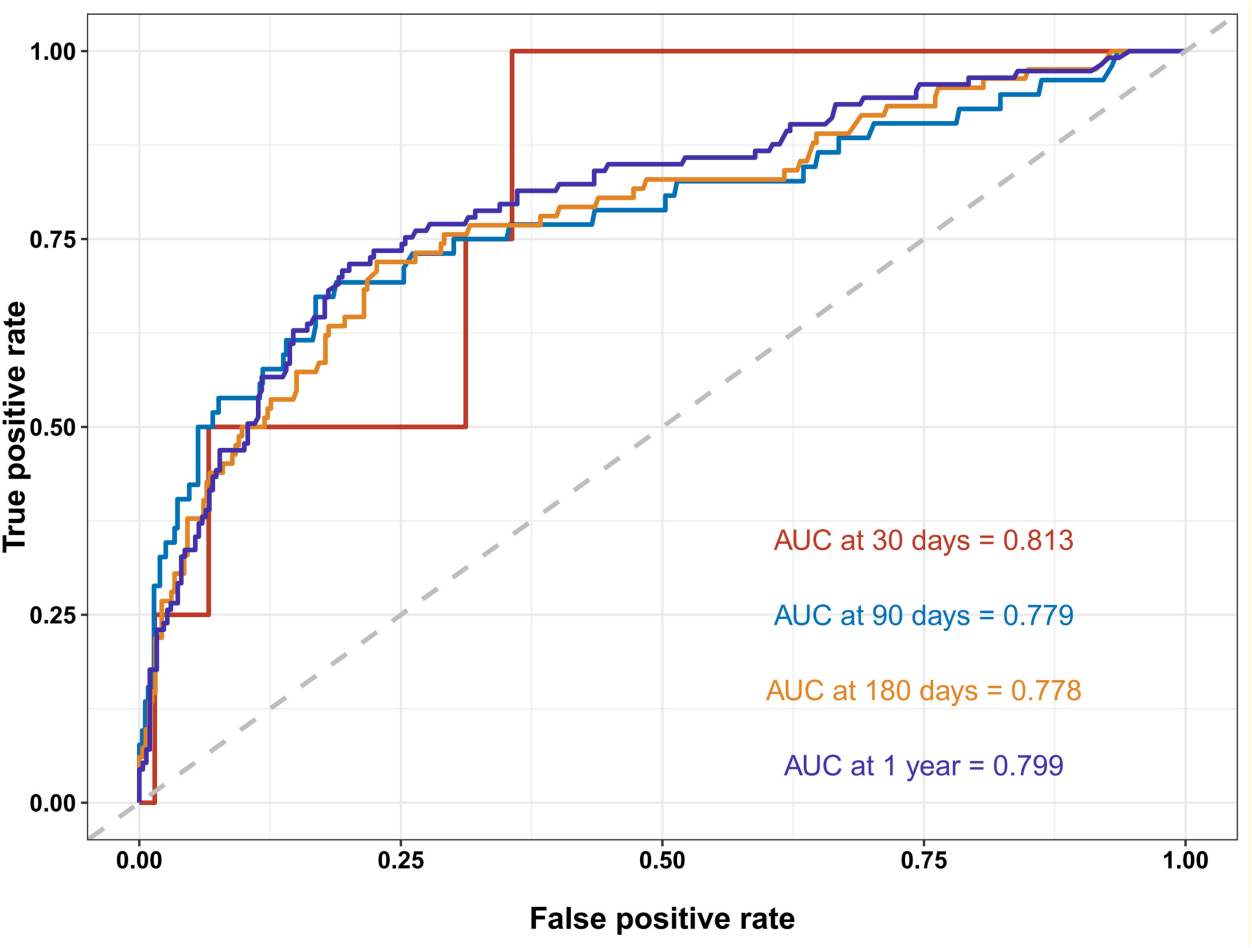
ROC curves for unplanned readmission of UC at 1, 3, 6 months, and 1 year. AUC, area under the curve. CAR showed consistently good discrimination across follow-up time points, with AUCs ranging from 0.778 to 0.813.

The AUC for predicting unplanned readmission using CAR was 0.813 at 1 month, 0.779 at 3 months, 0.778 at 6 months, and 0.799 at 1 year, indicating a robust predictive performance (Figure 4). In addition, we further evaluated the predictive performance of CRP alone and ALB alone for unplanned readmission in patients with UC. The AUCs of CRP at 1, 3, 6 months, and 1 year were 0.798, 0.771, 0.774, and 0.798, respectively (Supplementary Figure 4A), whereas the corresponding AUCs of ALB were 0.745, 0.706, 0.642, and 0.603, respectively (Supplementary Figure 4B).

## 4 Discussion

UC is a chronic inflammatory bowel disease characterized by symptoms such as diarrhea, abdominal pain, and hematochezia. UC typically presents with a fluctuating course, with patients frequently alternating between periods of remission and acute flare-ups. In the management of UC, unplanned readmission is a crucial indicator for assessing disease activity and treatment efficacy. This study is the first to establish a significant association between CAR and unplanned readmission in patients with UC. In the fully adjusted model, each unit increase in CAR was associated with a 126.9% increase in the risk of unplanned readmission. Compared to the lowest quartile (Q1), the highest quartile (Q4) of CAR was associated with an 11.38-fold increase in the risk of unplanned readmission (*P* < 0.001) (Table 2). This study also confirmed a non-linear relationship between CAR and unplanned readmission, revealing a pronounced dose-response effect that suggests that CAR serves as a reliable biomarker for predicting unplanned readmission.

Multiple studies have confirmed that CAR is closely associated with disease activity, endoscopic severity, acute severe status, and prognosis of biological therapy and surgery in UC. Ailing Liu et al. (21) found that CAR positively correlates with UC disease activity and endoscopic severity, demonstrating superior overall diagnostic performance compared to traditional serological markers such as CRP and ESR (AUC up to 0.918, optimal threshold approximately 0.06). In a large endoscopic grading cohort, CAR was significantly correlated with the Mayo Endoscopic Score (MES) and independently predicted moderate-to-severe endoscopic activity in multivariate models, particularly in patients with extensive colitis. However, its predictive value for mucosal healing and clinical remission is limited, suggesting that its primary utility lies in identifying and stratifying severe disease activity (17, 18). Header et al. (16) demonstrated that dynamic changes in CAR in Egyptian patients with acute severe ulcerative colitis (ASUC) have significant translational implications, with CAR ≥ 0.60 accurately identifying severe disease states (AUC ≈ 0.985). Con et al. (22) demonstrated that CAR measured on day 3 after infliximab (IFX) rescue therapy effectively stratified the 1-year risk of colectomy, outperforming traditional clinical scores (Mayo score, Travis score, Ho index) in multiple comparisons. Insufficient CAR decline indicates poor response to IFX and an increased risk of future surgical intervention.

Elevated CAR levels are closely associated with an increased risk of unplanned readmission in patients with UC, with potential biological mechanisms explained through three pathways: individual inflammatory responses, malnutrition, and compromised immunity. CRP, a non-specific inflammatory marker synthesized by the liver, reflects persistent systemic inflammation when elevated (23), indicating incomplete repair of the intestinal mucosal barrier (24). Decreased albumin levels result not only from chronic inflammation suppressing hepatic synthesis (25) but also correlate closely with intestinal protein loss and malnutrition (26, 27). Hypoalbuminemia weakens epithelial repair and immune defense functions, making patients more susceptible to infection and recurrent inflammation (28). Furthermore, the synergistic effects of inflammatory activation and reduced albumin levels may lead to immune dysfunction, such as impaired lymphocyte function and diminished antioxidant capacity.

Analysis using Kaplan-Meier survival curves demonstrated significant differences in unplanned readmission rates among UC patients grouped by quartiles of CAR, CRP, and ALB (log-rank test, *P* < 0.001). Further RCS regression analysis revealed a non-linear relationship between CAR and unplanned readmission. The study demonstrated a clear dose-response relationship between increasing CAR and unplanned readmission risk, with the risk increasing after CAR reached a certain threshold (0.654). Prior to the CAR inflection point (0.654), even a slight elevation significantly increased the risk of unplanned readmission, suggesting that imbalances in the inflammation-nutrition-immunity pathway may amplify adverse outcomes in patients with UC. Post-inflection, the risk increase flattened, potentially reflecting patients’ acute critical condition. Previous studies (21) reported CAR≈0.6 as a stratification threshold for severe illness. This demonstrates that the CAR effectively distinguishes risk groups, particularly exhibiting a high risk-identification capability within the moderate-to-severe range. For patients with extremely high CAR values, greater emphasis should be placed on post-discharge prognostic management and close follow-up rather than relying solely on sustained CAR elevation to assess risk gradients.

Subgroup analysis revealed that CAR significantly influenced unplanned readmissions in patients with hypertension (*P* = 0.011). Extensive research indicates hypertension correlates with inflammation and immune responses (29–32). This finding underscores the necessity of considering hypertension when evaluating the prognostic significance of CAR in patients with UC. Finally, ROC curve analysis revealed CAR’s strong predictive performance for unplanned readmission. The CAR demonstrated AUC values of 0.813, 0.779, 0.778, and 0.799 at the 1-month, 3-month, 6-month, and 1-year follow-up periods, respectively, indicating good-to-excellent predictive performance. In addition, we separately evaluated the independent predictive performance of CRP and ALB, and found that CRP showed relatively stable discriminative ability across follow-up time points, whereas the predictive performance of ALB gradually declined over time. Overall, CAR demonstrated the best predictive performance, with a more pronounced advantage for early readmission prediction (1–3 months). With longer follow-up, however, the incremental gain of CAR over CRP became smaller, possibly because CRP more consistently reflects inflammatory burden, whereas ALB is more susceptible to nutritional status, intestinal protein loss, and hemodilution due to intravenous fluid administration, thereby diminishing its contribution to longer-term readmission prediction (33–35).

This study is the first to establish a significant association between CAR and unplanned readmissions in UC patients. The efficacy of CAR as a predictive tool was comprehensively validated through Cox proportional hazards models, Kaplan-Meier survival curves, and RCS regression analysis. RCS analysis revealed a non-linear dose-response relationship between CAR and the risk of unplanned readmission, with a marked deceleration in risk increase when CAR reached 0.654, underscoring the importance of managing and monitoring high-risk patients with UC. Using ROC curve analysis, this study found that CAR demonstrated good predictive capability for unplanned readmission in both short-term (1 and 3 months) and long-term (6 months and 1 year) periods, with AUC values of 0.813, 0.779, 0.778, and 0.799, respectively. Additionally, subgroup analysis revealed a stronger association between CAR and unplanned readmission risk in patients with hypertension, further exploring the applicability of CAR in predicting unplanned readmission for UC across different populations.

## 5 Limitations

However, this study had certain limitations. First, as a retrospective study, it carries potential retrospective and selection biases. Future validation through prospective multicenter studies is needed to further confirm the efficacy of the CAR in predicting unplanned readmissions among patients with UC. Second, although this study considered multiple relevant variables affecting CAR’s predictive power for readmissions, some potential confounding factors may still exist. Therefore, future studies should design more comprehensive questionnaires and conduct prospective investigations to explore the relationship between the CAR and other relevant variables in greater depth. Finally, while this study confirmed that 95% of unplanned readmissions were due to UC reactivation, some patients were readmitted for other reasons. Consequently, future research should explore additional prognostic indicators to comprehensively evaluate the association between CAR and other potential factors.

## 6 Conclusion

The results of this study indicate that CAR exhibits a significant non-linear association and dose-response relationship with unplanned readmissions in patients with UC. After multivariate adjustment, CAR levels remained positively correlated with UC readmission rates in a non-linear manner. Furthermore, CAR demonstrated a strong predictive value for unplanned readmissions at 1 month, 3 month, 6 months, and 1 year. In summary, CAR serves as a novel biomarker for predicting unplanned readmissions in patients with UC, holding significant value for post-hospitalization management of these individuals.

## Data Availability

The original contributions presented in this study are included in this article/Supplementary material, further inquiries can be directed to the corresponding authors.

## References

[1] Le Berre C Honap S Peyrin-Biroulet L. Ulcerative colitis. *Lancet.* (2023) 402:571–84. 10.1016/s0140-6736(23)00966-2 37573077

[2] Voelker R. What is ulcerative colitis? *JAMA.* (2024) 331:716. 10.1001/jama.2023.23814 38306113

[3] Raine T Bonovas S Burisch J Kucharzik T Adamina M Annese V ECCO guidelines on therapeutics in ulcerative colitis: medical treatment. *J Crohns Colitis.* (2022) 16:2–17. 10.1093/ecco-jcc/jjab178 34635919

[4] Attauabi M Madsen G Bendtsen F Seidelin J Burisch J. Incidence, disease burden, and clinical presentation of patients newly diagnosed with inflammatory bowel disease in a population-based inception cohort. *J Crohns Colitis.* (2025) 19:jjae176. 10.1093/ecco-jcc/jjae176 39565294

[5] Kruger A Hinton A Afzali A. Index severity score and early readmission predicts increased mortality in ulcerative colitis patients. *Inflamm Bowel Dis.* (2019) 25:894–901. 10.1093/ibd/izy297 30247551

[6] Weissman S Sharma S Fung B Aziz M Sciarra M Swaminath A Increased mortality and healthcare costs upon hospital readmissions of ulcerative colitis flares: a large population-based cohort study. *Crohns Colitis 360.* (2021) 3:otab029. 10.1093/crocol/otab029 36776672 PMC9802231

[7] Miyatani Y Micic D. Revisiting the risk of hospital readmission in severe ulcerative colitis. *Inflamm Bowel Dis.* (2024) 30:688–9. 10.1093/ibd/izad212 37682866

[8] Dorofeyev A Holub S Babayeva G Ànaniin Î. Application of intellectual monitoring information technology in determining the severity of the condition of patients with inflammatory bowel diseases. *Wiad Lek.* (2021) 74:481–6. 10.36740/WLek202103118 33813454

[9] Dubinsky M Magro F Steinwurz F Hudesman D Kinnucan J Ungaro R Association of C-reactive protein and partial mayo score with response to tofacitinib induction therapy: results from the ulcerative colitis clinical program. *Inflamm Bowel Dis.* (2023) 29:51–61. 10.1093/ibd/izac061 35380664 PMC9825285

[10] Singh S Ananthakrishnan A Nguyen N Cohen B Velayos F Weiss J AGA clinical practice guideline on the role of biomarkers for the management of ulcerative colitis. *Gastroenterology.* (2023) 164:344–72. 10.1053/j.gastro.2022.12.007 36822736

[11] Nguyen G Du L Chong R Jackson T. Hypoalbuminaemia and postoperative outcomes in inflammatory bowel disease: the NSQIP surgical cohort. *J Crohns Colitis.* (2019) 13:1433–8. 10.1093/ecco-jcc/jjz083 31253985 PMC6821313

[12] Sofo L Caprino P Schena C Sacchetti F Potenza A Ciociola A. New perspectives in the prediction of postoperative complications for high-risk ulcerative colitis patients: machine learning preliminary approach. *Eur Rev Med Pharmacol Sci.* (2020) 24:12781–7. 10.26355/eurrev_202012_24178 33378027

[13] Nakamura N Honzawa Y Nishimon S Sano Y Tokutomi Y Ito Y Combined serum albumin, fecal immunochemical test, and leucine-rich alpha-2 glycoprotein levels for predicting prognosis in remitting patients with ulcerative colitis. *Sci Rep.* (2023) 13:13863. 10.1038/s41598-023-41137-x 37620642 PMC10449766

[14] Freitas M Capela T Macedo Silva V Arieira C Curdia Gonçalves T Dias de Castro F P051 identifying high-risk patients with acute severe ulcerative colitis: is the ACE index useful? *Am J Gastroenterol.* (2021) 116:S13. 10.14309/01.ajg.0000798804.53697.05

[15] Mai R Lu T Lu R Zeng C Lian F Li L C-Reactive protein-albumin ratio (CAR): a more promising inflammation-based prognostic marker for patients undergoing curative hepatectomy for hepatocellular carcinoma. *J Inflamm Res.* (2024) 17:919–31. 10.2147/JIR.S441623 38370468 PMC10871143

[16] Header D Aboelwafa R Elkeleny M Bedewy E Ellakany AI. C-reactive protein/albumin ratio (CAR) as a marker for detecting acute severe ulcerative colitis in Egyptian patients. *Rev Gastroenterol Mex.* (2022) 87:447–54. 10.1016/j.rgmxen.2022.06.007 35810089

[17] Cui J Li X Zhang Z Gao H Li J. Common laboratory blood test immune panel markers are useful for grading ulcerative colitis endoscopic severity. *BMC Gastroenterol.* (2022) 22:540. 10.1186/s12876-022-02634-x 36572872 PMC9791766

[18] Furukawa S Yagi S Shiraishi K Miyake T Tange K Hashimoto Y Effect of disease duration on the association between C-reactive protein-albumin ratio and endoscopic activity in ulcerative colitis. *BMC Gastroenterol.* (2022) 22:39. 10.1186/s12876-022-02113-3 35094678 PMC8802502

[19] Rubin D Ananthakrishnan A Siegel C Barnes E Long MD. ACG clinical guideline update: ulcerative colitis in adults. *Am J Gastroenterol.* (2025) 120:1187–224. 10.14309/ajg.0000000000003463 40701556

[20] Conilione P Jessup R Gust A. Novel machine learning model for predicting multiple unplanned hospitalisations. *BMJ Health Care Inform.* (2023) 30:e100682. 10.1136/bmjhci-2022-100682 37015761 PMC10083802

[21] Liu A Lv H Tan B Shu H Yang H Li J Accuracy of the highly sensitive C-reactive protein/albumin ratio to determine disease activity in inflammatory bowel disease. *Medicine.* (2021) 100:e25200. 10.1097/md.0000000000025200 33832080 PMC8036110

[22] Con D Andrew B Nicolaides S van Langenberg D Vasudevan A. Biomarker dynamics during infliximab salvage for acute severe ulcerative colitis: C-reactive protein (CRP)-lymphocyte ratio and CRP-albumin ratio are useful in predicting colectomy. *Intest Res.* (2022) 20:101–13. 10.5217/ir.2020.00146 33902267 PMC8831766

[23] Bakkaloglu O Sen G Kepil N Eskazan T Kurt E Onal U Comparative value of CRP and FCP for endoscopic and histologic remissions in ulcerative colitis. *Diagnostics.* (2024) 14:2283. 10.3390/diagnostics14202283 39451607 PMC11506680

[24] Shen M Shi Y Ge Z Qian J. Effects of mesalamine combined with live combined bifidobacterium, Lactobacillus and enterococcus capsules on intestinal mucosa barrier function and intestinal microbiota in mildly active Crohn’s disease patients. *J Invest Surg.* (2024) 37:2297565. 10.1080/08941939.2023.2297565 38159563

[25] Casulleras M Flores-Costa R Duran-Güell M Zhang I López-Vicario C Curto A Albumin lipidomics reveals meaningful compositional changes in advanced cirrhosis and its potential to promote inflammation resolution. *Hepatol Commun.* (2022) 6:1443–56. 10.1002/hep4.1893 35178899 PMC9134813

[26] Zheng J Zhang X Zhang L Li L Chen M Chen R Serum albumin and its trajectory are associated with therapeutic outcomes in ulcerative colitis. *Clin Gastroenterol Hepatol.* (2025) 23:1808–16. 10.1016/j.cgh.2024.10.036 39694206

[27] Massironi S Viganò C Palermo A Pirola L Mulinacci G Allocca M Inflammation and malnutrition in inflammatory bowel disease. *Lancet Gastroenterol Hepatol.* (2023) 8:579–90. 10.1016/s2468-1253(23)00011-0 36933563

[28] Wiedermann C. Hypoalbuminemia as surrogate and culprit of infections. *Int J Mol Sci.* (2021) 22:4496. 10.3390/ijms22094496 33925831 PMC8123513

[29] Zhang Z Zhao L Zhou X Meng X Zhou X. Role of inflammation, immunity, and oxidative stress in hypertension: new insights and potential therapeutic targets. *Front Immunol.* (2022) 13:1098725. 10.3389/fimmu.2022.1098725 36703963 PMC9871625

[30] Guzik T Touyz R. Oxidative stress, inflammation, and vascular aging in hypertension. *Hypertension.* (2017) 70:660–7. 10.1161/hypertensionaha.117.07802 28784646

[31] Aboukhater D Morad B Nasrallah N Nasser S Sahebkar A Kobeissy F Inflammation and hypertension: underlying mechanisms and emerging understandings. *J Cell Physiol.* (2023) 238:1148–59. 10.1002/jcp.31019 37039489

[32] Harrison D Coffman T Wilcox C. Pathophysiology of hypertension: the mosaic theory and beyond. *Circ Res.* (2021) 128:847–63. 10.1161/circresaha.121.318082 33793328 PMC8023760

[33] Ishida N Takebe T Takahashi K Asai Y Matsuura T Yamade M Optimal positioning of biomarkers according to ulcerative colitis activity. *Sci Rep.* (2025) 15:19916. 10.1038/s41598-025-04908-2 40481096 PMC12144207

[34] Allison S Lobo D. The clinical significance of hypoalbuminaemia. *Clin Nutr.* (2024) 43:909–14. 10.1016/j.clnu.2024.02.018 38394971

[35] Levitt D Levitt M. Protein losing enteropathy: comprehensive review of the mechanistic association with clinical and subclinical disease states. *Clin Exp Gastroenterol.* (2017) 10:147–68. 10.2147/CEG.S136803 28761367 PMC5522668

