# Supplementary material for: Association between the C-reactive protein to albumin ratio and unplanned readmission in ulcerative colitis: insights from a cohort study
Source: Front Med (Lausanne). 2026 Feb 11;13:1715011. doi: 10.3389/fmed.2026.1715011 (PMC12932606; doi:10.3389/fmed.2026.1715011)
Supplement: Supplementary file 1 [file Supplementary_file_1.docx]

Supplementary Material

Association Between the C-reactive protein to albumin ratio and Unplanned Readmission in Ulcerative Colitis: Insights from a Cohort Study

**
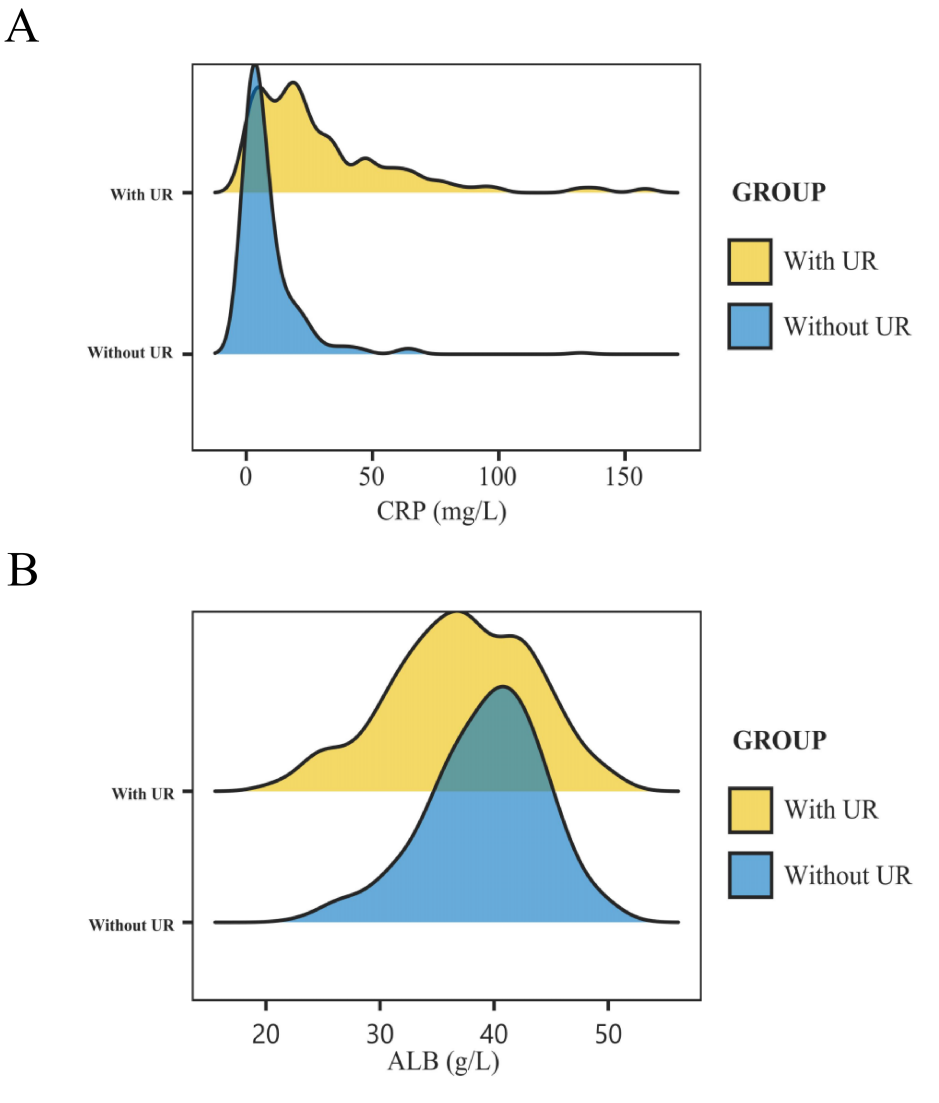
**

**Supplementary figure** **1.** Density maps of CRP and ALB in the study cohort. A: Distribution of CRP; B: Distribution of ALB.

CRP, C-reactive protein; ALB, albumin; UR, unplanned readmission.


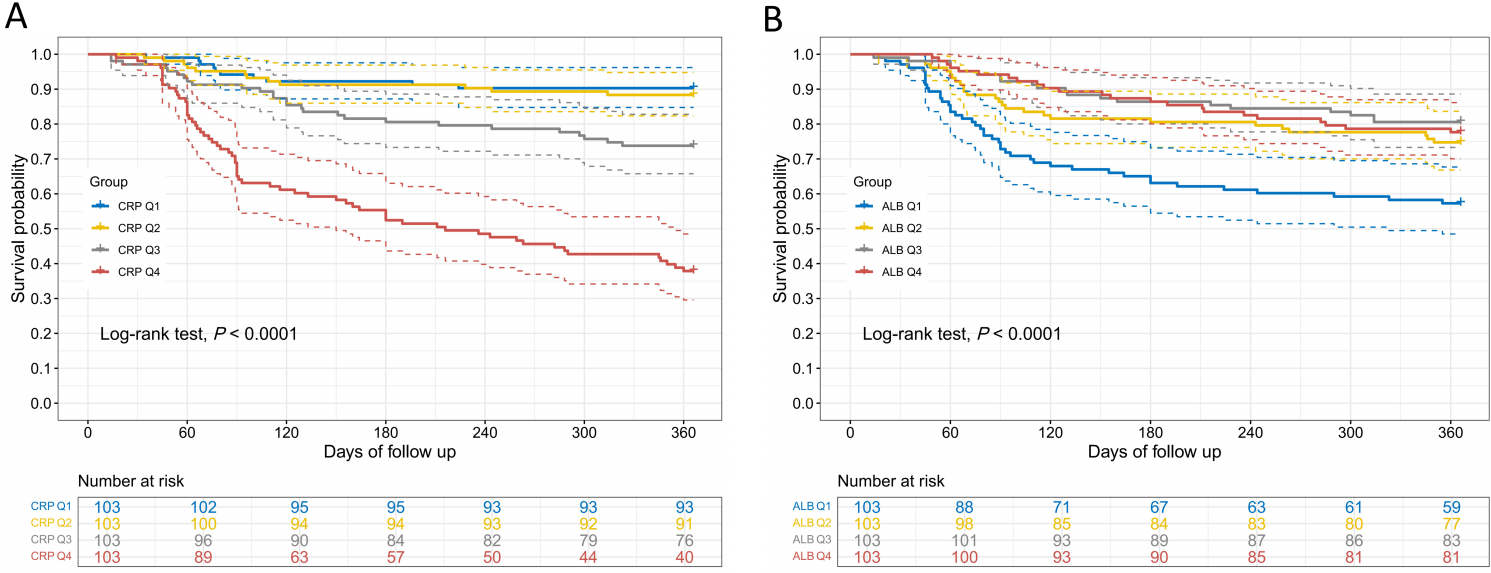


**Supplementary Figure 2.** Kaplan–Meier curves of unplanned readmission in ulcerative colitis. A: By CRP quartiles; B: By ALB quartiles.

CRP, C-reactive protein; ALB, albumin.


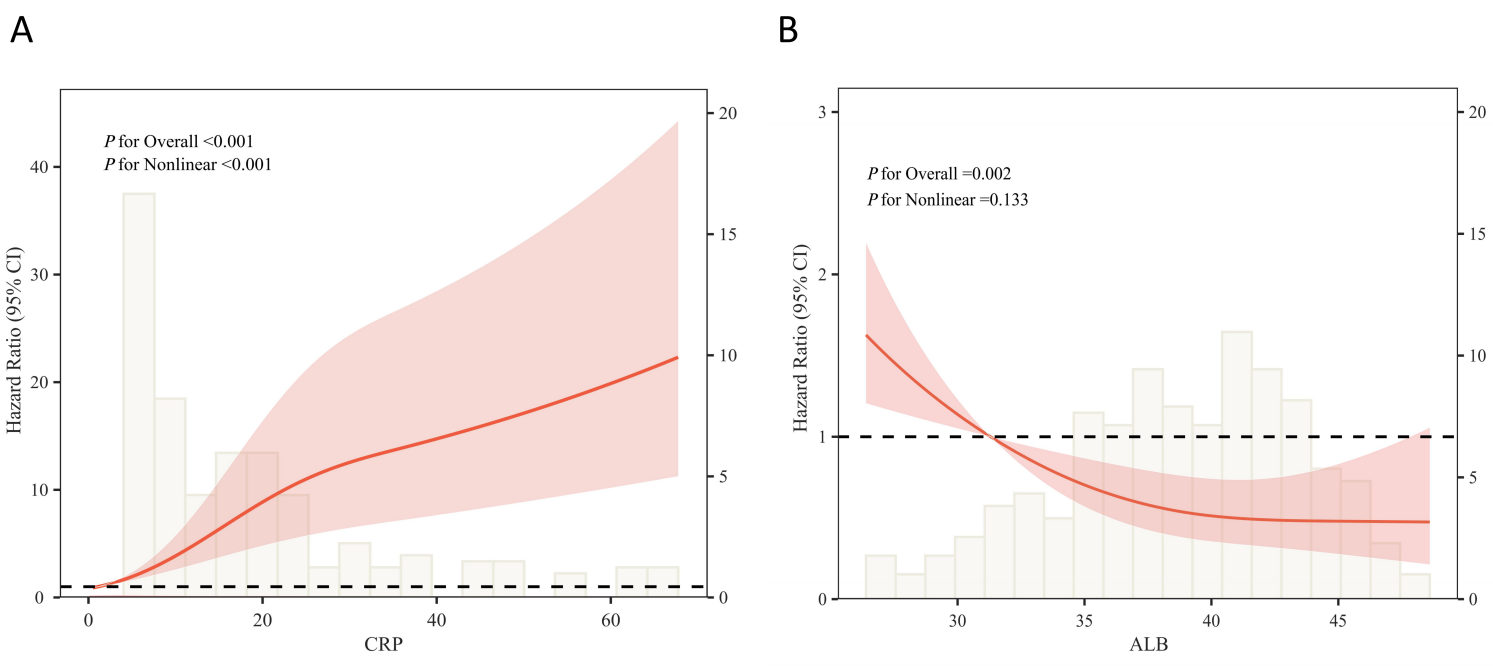


**Supplementary figure** **3.** Three-fold spline analysis of CRP and ALB with one-year unplanned readmission risk in ulcerative colitis. A: CRP and readmission risk; B: ALB and readmission risk.

CRP, C-reactive protein; ALB, albumin.


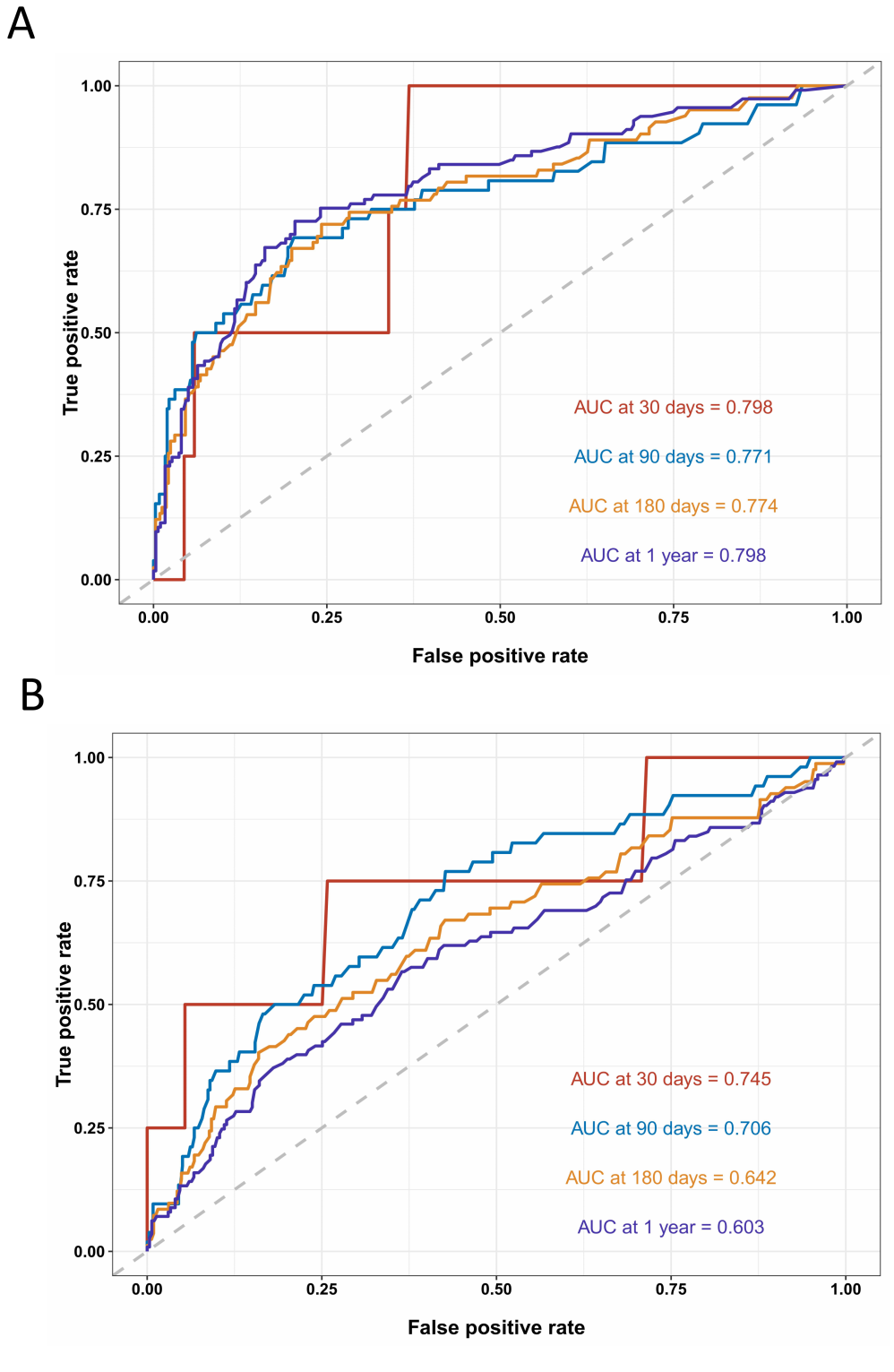


**Supplementary figure 4.** ROC curves of CRP and ALB for predicting unplanned readmission in ulcerative colitis.A: ROC curves of CRP for predicting unplanned readmission at 30 days, 90 days, 180 days, and 1 year ; B: ROC curves of ALB for predicting unplanned readmission at 30 days, 90 days, 180 days, and 1 year.

CRP, C-reactive protein; ALB, albumin; AUC, area under the curve.

**Supplementary table 1** Baseline Characteristics

| Characteristics | Total | Unplanned Readmission | | *P* value |
| --- | --- | --- | --- | --- |
|  | （N=412） | （With UR=299） | （Without UR=113) |  |
| Age (yr) | 46.59 ± 13.78 | 46.70 ± 13.95 | 46.32 ± 13.38 | 0.801 |
| Sex, *n* (%) |  |  |  | 0.611 |
| Female | 247 (59.95) | 177 (59.20) | 70 (61.95) |  |
| Male | 165 (40.05) | 122 (40.80) | 43 (38.05) |  |
| Smoke, *n* (%) |  |  |  | 0.195 |
| No | 367 (89.08) | 270 (90.30) | 97 (85.84) |  |
| Yes | 45 (10.92) | 29 (9.70) | 16 (14.16) |  |
| Alcohol, *n* (%) |  |  |  | 0.909 |
| No | 373 (90.53) | 271 (90.64) | 102 (90.27) |  |
| Yes | 39 (9.47) | 28 (9.36) | 11 (9.73) |  |
| Hypertension, *n* (%) |  |  |  | 0.66 |
| No | 376 (91.26) | 274 (91.64) | 102 (90.27) |  |
| Yes | 36 (8.74) | 25 (8.36) | 11 (9.73) |  |
| Heart disease, *n* (%) |  |  |  | 0.824 |
| No | 396 (96.12) | 287 (95.99) | 109 (96.46) |  |
| Yes | 16 (3.88) | 12 (4.01) | 4 (3.54) |  |
| Diabetes, *n* (%) |  |  |  | 0.601 |
| No | 397 (96.36) | 289 (96.66) | 108 (95.58) |  |
| Yes | 15 (3.64) | 10 (3.34) | 5 (4.42) |  |
| Use of 5-ASA, *n* (%) |  |  |  | 0.084 |
| No | 81 (19.66) | 65 (21.74) | 16 (14.16) |  |
| Yes | 331 (80.34) | 234 (78.26) | 97 (85.84) |  |
| Use of GCS, *n* (%) |  |  |  | 0.001 |
| No | 324 (78.64) | 247 (82.61) | 77 (68.14) |  |
| Yes | 88 (21.36) | 52 (17.39) | 36 (31.86) |  |
| Use of immunosuppressive agents, *n* (%) |  |  |  | 0.824 |
| No | 396 (96.12) | 287 (95.99) | 109 (96.46) |  |
| Yes | 16 (3.88) | 12 (4.01) | 4 (3.54) |  |
| Use of biologics, *n* (%) |  |  |  | <0.001 |
| No | 395 (95.9) | 297（99.3） | 98（86.7） |  |
| Yes | 17 ( 4.1) | 2（0.7） | 15（13.3） |  |
| Use of probiotics, *n* (%) |  |  |  | 0.384 |
| No | 233 (56.55) | 173 (57.86) | 60 (53.10) |  |
| Yes | 179 (43.45) | 126 (42.14) | 53 (46.90) |  |
| Clinical typing, *n* (%) |  |  |  | 0.316 |
| Initial hairstyle | 59 (14.32) | 46 (15.38) | 13 (11.50) |  |
| Recurrent type | 353 (85.68) | 253 (84.62) | 100 (88.50) |  |
| Extent of disease, *n* (%) |  |  |  | 0.019 |
| E1 | 120 (29.13) | 93 (31.10) | 27 (23.89) |  |
| E2 | 140 (33.98) | 108 (36.12) | 32 (28.32) |  |
| E3 | 152 (36.89) | 98 (32.78) | 54 (47.79) |  |
| Colonoscopy examination, *n* (%) |  |  |  | 0.048 |
| Relief period | 22 (5.34) | 20 (6.69) | 2 (1.77) |  |
| Active phase | 390 (94.66) | 279 (93.31) | 111 (98.23) |  |
| Mucosal biopsy, *n* (%) |  |  |  | <0.001 |
| normal | 7 (1.70) | 7 (2.34) | 0 (0.00) |  |
| mild | 110 (26.70) | 94 (31.44) | 16 (14.16) |  |
| moderate | 206 (50.00) | 145 (48.49) | 61 (53.98) |  |
| severe | 89 (21.60) | 53 (17.73) | 36 (31.86) |  |
| Hematochezia condition, *n* (%) |  |  |  | 0.005 |
| normal | 55 (13.35) | 39 (13.04) | 16 (14.16) |  |
| mild | 125 (30.34) | 102 (34.11) | 23 (20.35) |  |
| moderate | 186 (45.15) | 133 (44.48) | 53 (46.90) |  |
| severe | 46 (11.17) | 25 (8.36) | 21 (18.58) |  |
| Increased defecation, *n* (%) |  |  |  | <0.001 |
| normal | 82 (19.90) | 74 (24.75) | 8 (7.08) |  |
| mild | 106 (25.73) | 82 (27.42) | 24 (21.24) |  |
| moderate | 113 (27.43) | 85 (28.43) | 28 (24.78) |  |
| severe | 111 (26.94) | 58 (19.40) | 53 (46.90) |  |
| Platelet count (×10^9^ /L) | 266.00 (119.00) | 262.00 (110.00) | 275.00 (139.00) | 0.015 |
| Hemoglobin (g/L) | 128.50 (28.00) | 129.00 (26.00) | 128.00 (37.00) | 0.132 |
| White blood cell count (×10^9^ /L) | 6.15 (3.11) | 5.94 (2.78) | 6.55 (4.63) | 0.013 |
| Alanine minotransferase (U/L) | 13.00 (11.00) | 14.00 (11.00) | 13.00 (12.00) | 0.416 |
| Aspartate aminotransferase (U/L) | 17.00 (9.00) | 17.00 (8.00) | 16.00 (8.00) | 0.023 |
| Total bilirubin (μmoI/L) | 10.80 (5.80) | 10.80 (6.20) | 10.80 (5.10) | 0.102 |
| Albumin (g/L) | 38.67 ±5.46 | 39.24 ± 5.05 | 37.15 ± 6.19 | 0.002 |
| C-reactive protein (mg/L) | 6.00 (15.85) | 4.00 (7.40) | 19.70 (27.00) | <0.001 |
| C-reactive protein to albumin ratio | 0.16 (0.40) | 0.11 (0.20) | 0.55 (0.89) | <0.001 |

UR, unplanned readmission; 5-ASA, 5-aminosalicylic acid; GCS, glucocorticoids.

**Supplementary table 2** The final Cox proportional hazards model assessing the association between CAR and Unplanned Readmission in Ulcerative Colitis.

| Characteristics | Number (%) | HR (95%CI) | *P* value |
| --- | --- | --- | --- |
| Age (yr) | 46.59 (13.78) | 1.01(1.00, 1.03) | 0.175 |
| Sex |  |  | 0.378 |
| Female | 247 (60.0) |  |  |
| Male | 165 (40.0) | 1.22(0.78, 1.91) |  |
| Smoke |  |  | 0.001 |
| No | 367 (89.1) |  |  |
| Yes | 45 (10.9) | 3.39(1.65, 6.98) |  |
| Alcohol |  |  | 0.167 |
| No | 373 (90.5) |  |  |
| Yes | 39 ( 9.5) | 0.56(0.25, 1.27) |  |
| Diabetes |  |  | 0.527 |
| No | 397 (96.4) |  |  |
| Yes | 15 ( 3.6) | 0.73(0.27, 1.54) |  |
| Heart disease |  |  | 0.294 |
| No | 396 (96.1) |  |  |
| Yes | 16 ( 3.9) | 0.55(0.18, 1.68) |  |
| Hypertension |  |  | 0.26 |
| No | 376 (91.3) |  |  |
| Yes | 36 ( 8.7) | 1.52(0.73, 3.16) |  |
| Use of 5-ASA |  |  | 0.818 |
| No | 81 (19.7) |  |  |
| Yes | 331 (80.3) | 0.93(0.52, 1.68) |  |
| Use of biologics |  |  | 0 |
| No | 395 (95.9) |  |  |
| Yes | 17 ( 4.1) | 11.18(5.84, 21.41) |  |
| Use of GCS |  |  | 0.064 |
| No | 324 (78.6) |  |  |
| Yes | 88 (21.4) | 1.53(0.98, 2.41) |  |
| Use of immunosuppressive agents |  |  | 0.647 |
| No | 396 (96.1) |  |  |
| Yes | 16 ( 3.9) | 0.78(0.27, 2.26) |  |
| Use of probiotics |  |  | 0.367 |
| No | 233 (56.6) |  |  |
| Yes | 179 (43.4) | 0.83(0.55, 1.25) |  |
| Platelet count (×10^9^ /L) | 282.07 (106.03) | 1(1.00, 1.00) | 0.811 |
| White blood cell count (×10^9^ /L) | 6.62 (2.61) | 0.99(0.92, 1.07) | 0.859 |
| Alanine aminotransferase (U/L) | 17.47 (13.71) | 1.01(0.99, 1.03) | 0.373 |
| Aspartate aminotransferase (U/L) | 18.81 (10.20) | 0.99(0.96, 1.02) | 0.388 |
| Hemoglobin (g/L) | 125.04 (23.47) | 0.99(0.98, 1.00) | 0.217 |
| Total bilirubin (μmoI/L) | 12.02 (5.16) | 0.99(0.95, 1.03) | 0.624 |
| C-reactive protein to albumin ratio |  |  |  |
| 0 | 103 (25.0) |  |  |
| 1 | 103 (25.0) | 1.32(0.53, 3.24) | 0.552 |
| 2 | 103 (25.0) | 3.14(1.40, 7.04) | 0.005 |
| 3 | 103 (25.0) | 11.38(5.21, 24.88) | 0 |

5-ASA, 5-aminosalicylic acid; GCS, glucocorticoids.
